# Supplementary material for: Identification of a highly conserved neutralizing epitope within the RBD region of diverse SARS-CoV-2 variants
Source: Nat Commun. 2024 Jan 29;15:842. doi: 10.1038/s41467-024-45050-3 (PMC10825162; doi:10.1038/s41467-024-45050-3)
Supplement: Supplementary file 1 — Supplementary Information [file 41467_2024_45050_MOESM1_ESM.pdf]

Supplementary Materials for

Identification of a highly conserved neutralizing epitope within the RBD region of diverse SARS-CoV-2 variants

Yanqun Wang[1,4,10,11], An Yan[2,11], Deyong Song[3,11], Maoqin Duan[5,11], Chuangchuang Dong[3], Jiantao Chen[1], Zihe Jiang[2], Yuanzhu Gao[2], Muding Rao[3], Jianxia Feng[3], Zhaoyong Zhang[1], Ruxi Qi[2], Xiaomin Ma[2], Hong Liu[3], Beibei Yu[3], Qiaoping Wang[3], Mengqi Zong[3], Jie Jiao[3], Pingping Xing[3], Rongrong Pan[3], Dan Li[3], Juxue Xiao[1], Junbo Sun[3], Ying Li[3], Linfeng Zhang[3], Zhenduo Shen[3], Baiping Sun[3], Yanyan Zhao[3], Lu Zhang[6], Jun Dai[6], Jingxian Zhao[1], Lan Wang[5*], Changlin Dou[3*], Zheng Liu[2*], Jincun Zhao[1,7,8,9*]

Author affiliations

[1]State Key Laboratory of Respiratory Disease, National Clinical Research Center for Respiratory Disease, Guangzhou Institute of Respiratory Health, the First Affiliated Hospital of Guangzhou Medical University, Guangzhou, China

[2]Cryo-electron Microscopy Center, Southern University of Science and Technology, Shenzhen, China

[3]Antibody Research and Development Center, Shandong Boan Biotechnology Co., Ltd., Yantai, China

[4]Clinical Laboratory Medicine Department, The Second Affiliated Hospital of Guangzhou Medical University, Guangzhou, China

[5]Division of Monoclonal Antibodies, Institute for Biological Product Control, National Institutes for Food and Drug Control (NIFDC), Beijing, China

[6]Health and Quarantine Laboratory, Guangzhou Customs District Technology Centre, Guangzhou, China

[7]Guangzhou Laboratory, Bio-Island, Guangzhou, China

[8]Shanghai Institute for Advanced Immunochemical Studies, School of Life Science and Technology, ShanghaiTech University, Shanghai, China

[9]Institute for Hepatology, National Clinical Research Center for Infectious Disease, Shenzhen Third People’s Hospital; The Second Affiliated Hospital, School of Medicine, Southern University of Science and Technology, Shenzhen, China.
[10]GMU-GIBH Joint School of Life Sciences, Guangzhou Medical University, Guangzhou, China

[11]These authors contributed equally;
*Correspondence author: Jincun Zhao (zhaojincun@gird.cn), Zheng Liu (liuz3@sustech.edu.cn), Changlin Dou (douchanglin@boan-bio.com), Lan Wang (wanglan@nifdc.org.cn)

This PDF file includes:

Supplementary Figures. 1-15

Supplementary Tables 1-6

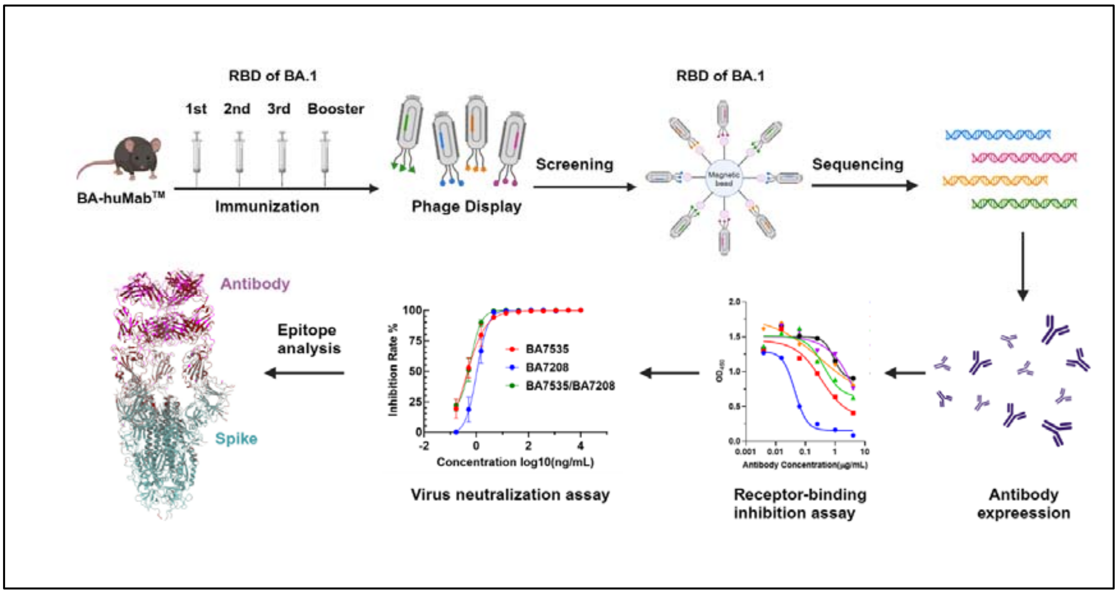

Supplementary Figure. 1. Graphical Abstract for anti-SARS-CoV-2 antibody identification and characterization. mAbs against SARS-CoV-2 variants were produced through immunizing six human antibody transgenic mice BA-huMab with RBD proteins of the SARS-CoV-2 BA.1 variant followed by phage display. ScFvs of picked clones were sequenced and then converted into IgG1 format for expression. Antibodies showing RBD/ACE2 receptor blocking activity were obtained for in vitro characterization. Structure of BA.2 Spike trimer with BA7535-Fab was determined (created using BioRender: https://biorender.com/).

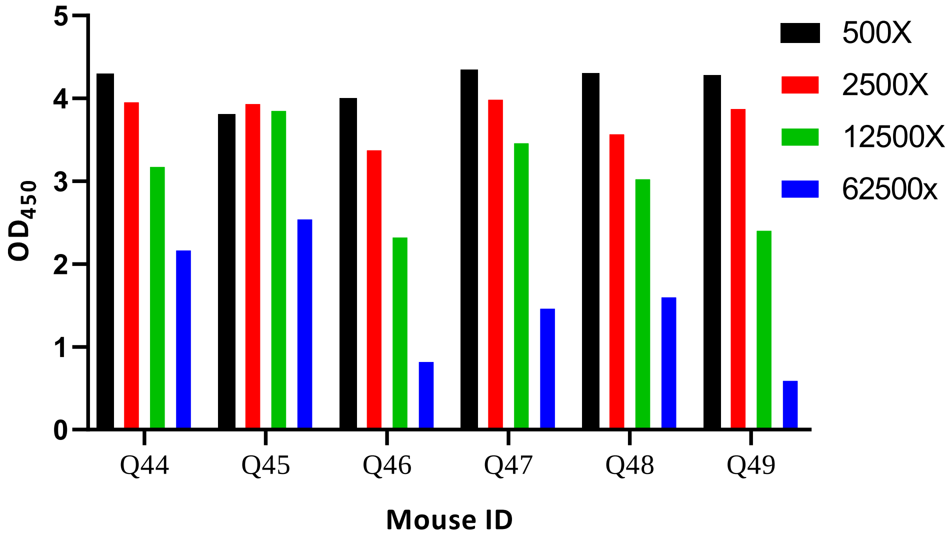

Supplementary Figure. 2. Antibody production in six human antibody transgenic mice. Six transgenic mice Q44-Q49 were immunized and boosted with BA.1 RBD protein, serial diluted sera (500-fold, 2500-fold, 12500-fold, 62500-fold) were used for RBD-specific antibody titer determination by ELISA. Source data are provided as a Source Data file.

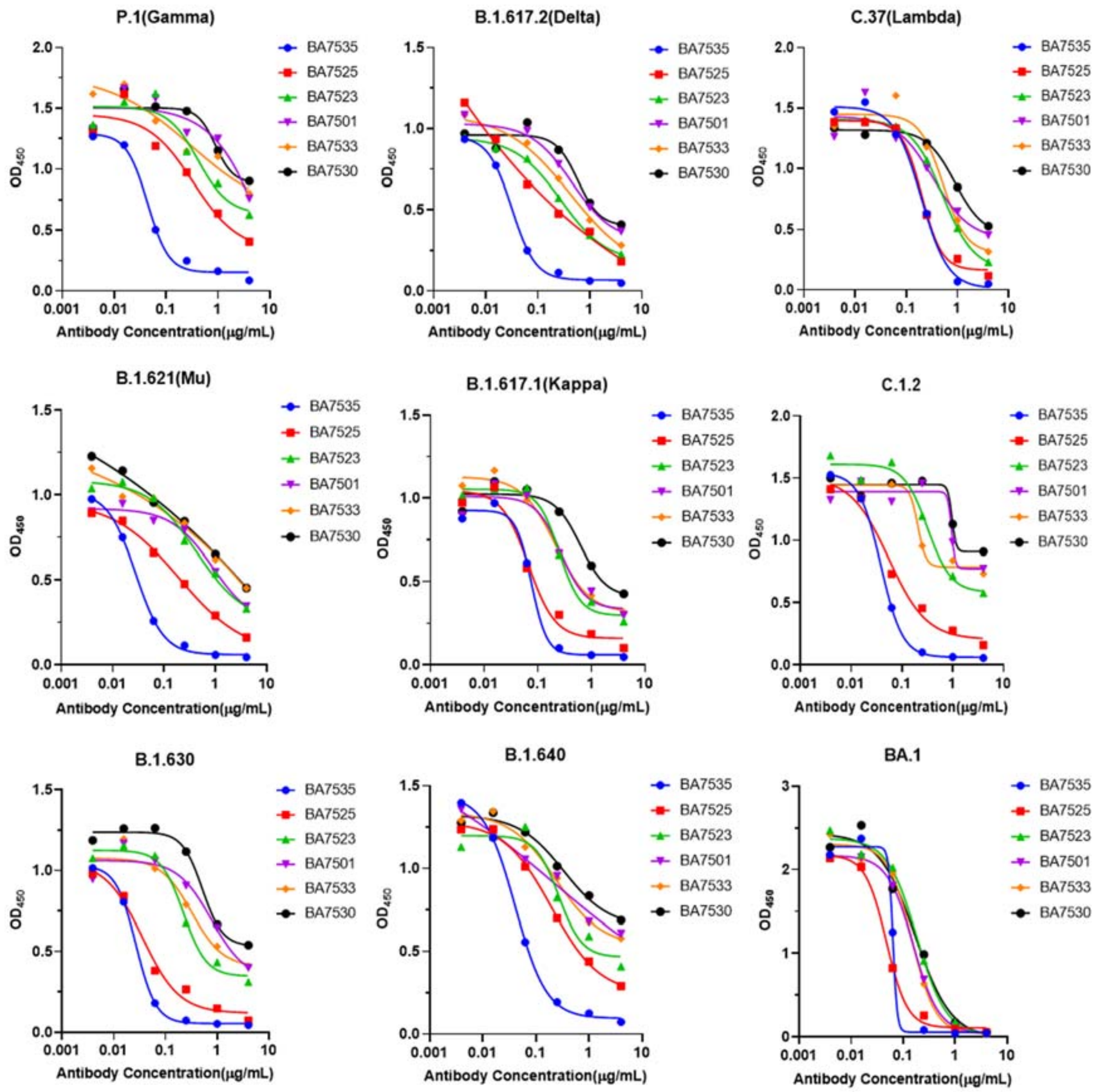

Supplementary Figure. 3. Blocking activity curves of potential candidates against RBDs of 9 SARS-CoV-2 variants. Blocking activity of the six mAbs against RBDs of the 9 SARS-CoV-2 variants (B.1.621, BA.1, C.1.2, B.1.640, P.1, C.37, B.1.617.1, B.1.630 and B.1.617.2) were detected in ELISA, respectively. Source data are provided as a Source Data file.

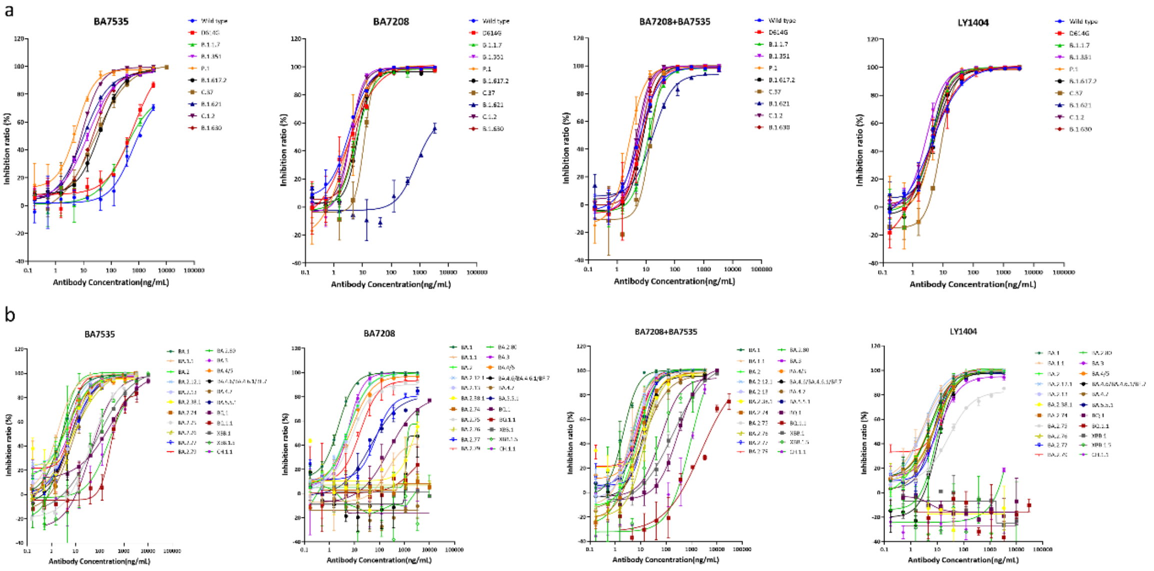

Supplementary Figure. 4. Neutralization curves of anti-SARS-CoV-2 antibodies against previous and new emerging SARS-CoV-2 variants. Neutralization curves of of BA7535, BA7208, their combination BA7535/BA7208 and LY-COV1404 against **(a)** previous and **(b)** new emerging Omicron variants in a pseudovirus system were showed respectively. Two biological replicates were performed and represented as Mean ± SD. Source data are provided as a Source Data file.

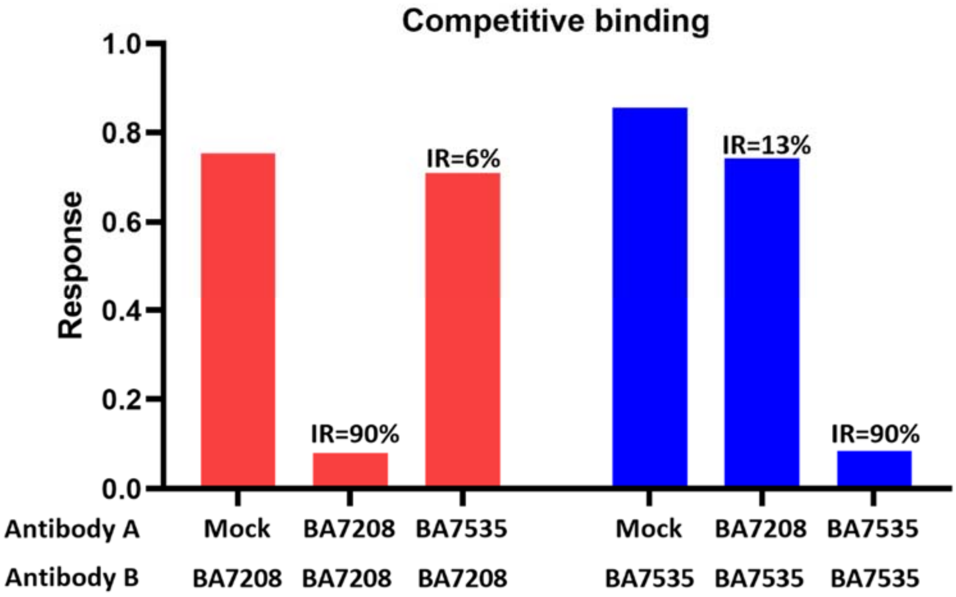

Supplementary Figure. 5. Competitive binding of the antibodies (BA7208 and BA7535) in BLI based competitive binding assay. Competitive binding of the antibodies was performed on a ForteBio Octet Red96 system. The sensors loaded RBD protein were saturated with the first antibody (antibody A), then subjected to the second antibody (antibody B). Inhibitory rate (IR) was shown as a percentage. Define >50% IR against antibody B means antibody A strongly competes with antibody B to bind the RBD. Source data are provided as a Source Data file.

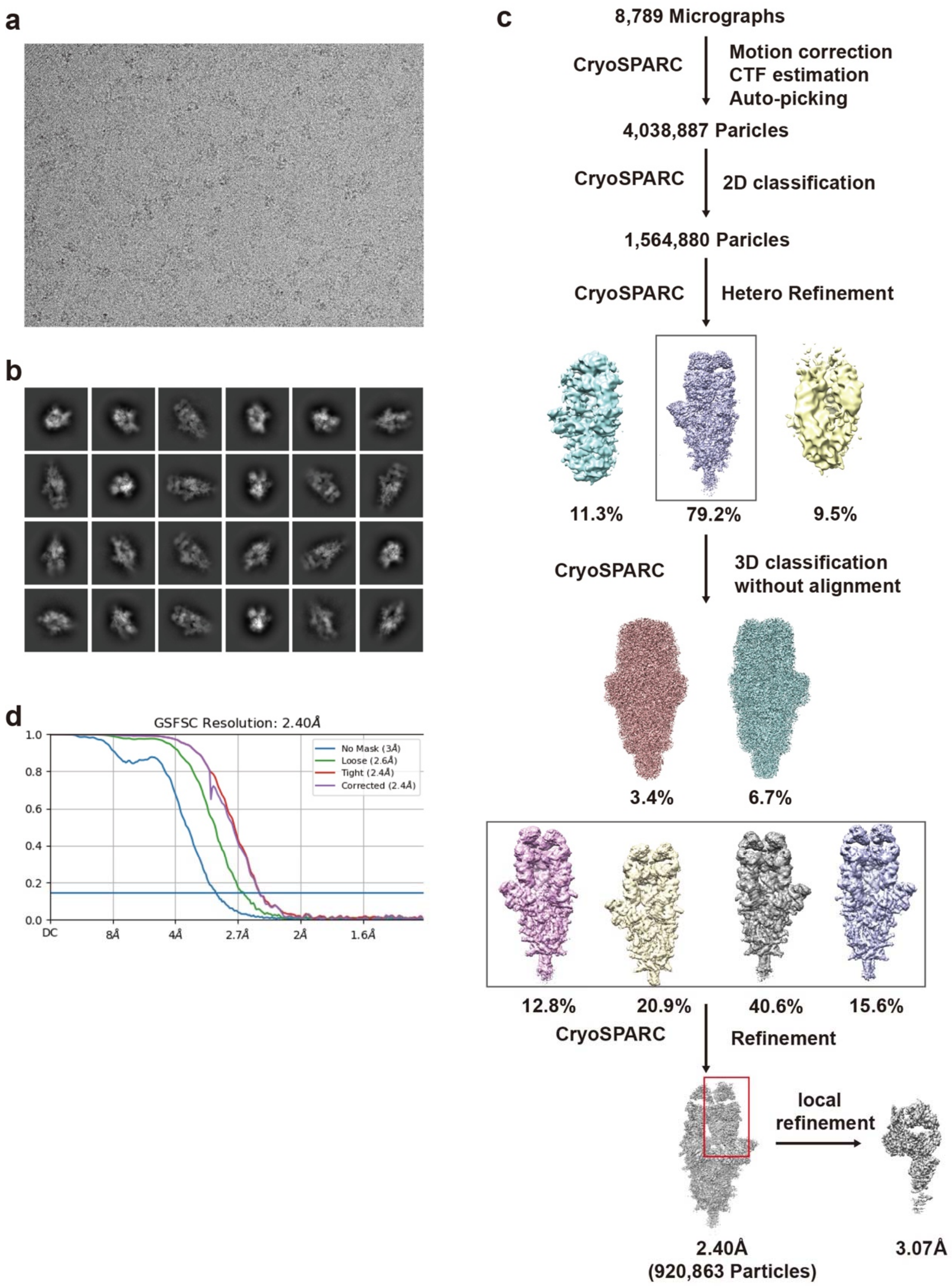

Supplementary Figure. 6. Workflow of Cryo-EM data collection and image processing of Omicron BA.2 Spike/BA7535-Fab.

(a) Representative cryo-EM micrograph of Omicron BA.2-Spike/BA7535. **(b)** 2D class average images of Omicron BA.2 -Spike/BA7535-Fab. **(c)** Workflow of cryo-EM image processing and reconstruction. The global map is colored by results of local resolution estimation. **(d)** the FSC curves of the reconstructions.

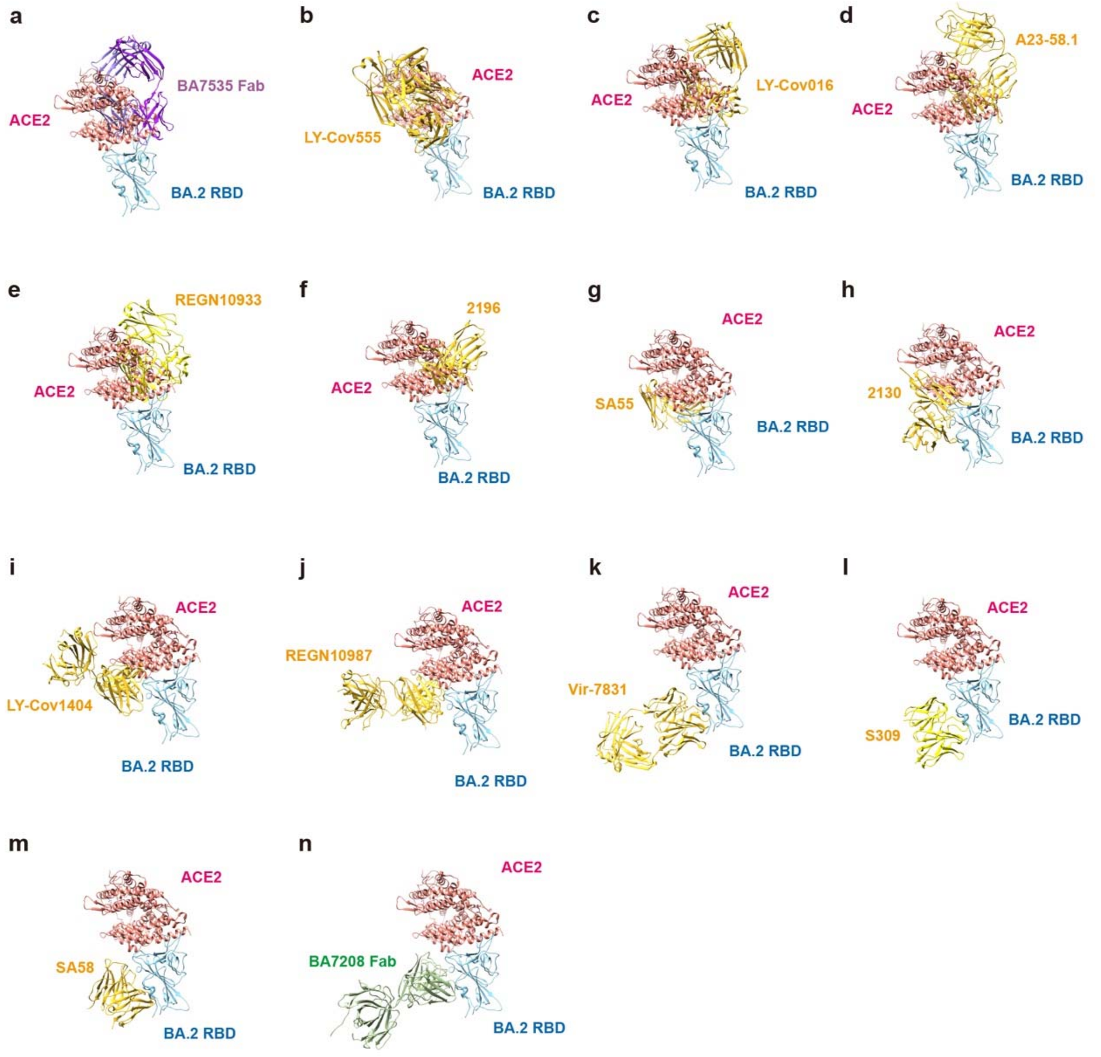

Supplementary Figure. 7. BA7535 binding mode analysis compared to antibodies that are licensed for clinical use.

The complex of BA.2 RBD (cyan) and ACE2 (pink) is superimposed with antibodies: **(a)** BA7535-Fab (purple), **(b)** LY-Cov555 (yellow), **(c)** LY-Cov016, **(d)** A23-58.1 (yellow), **(e)** REGN10933 (yellow), **(f)** 2196 (yellow), **(g)** SA55 (yellow), **(h)** 2130 (yellow), **(i)** LY-Cov-1404 (yellow), **(j)** REGN10987 (yellow), **(k)** Vir-7831 (yellow), **(l)** S309 (yellow), **(m)** SA58 (yellow), and **(n)** BA7208 (green).

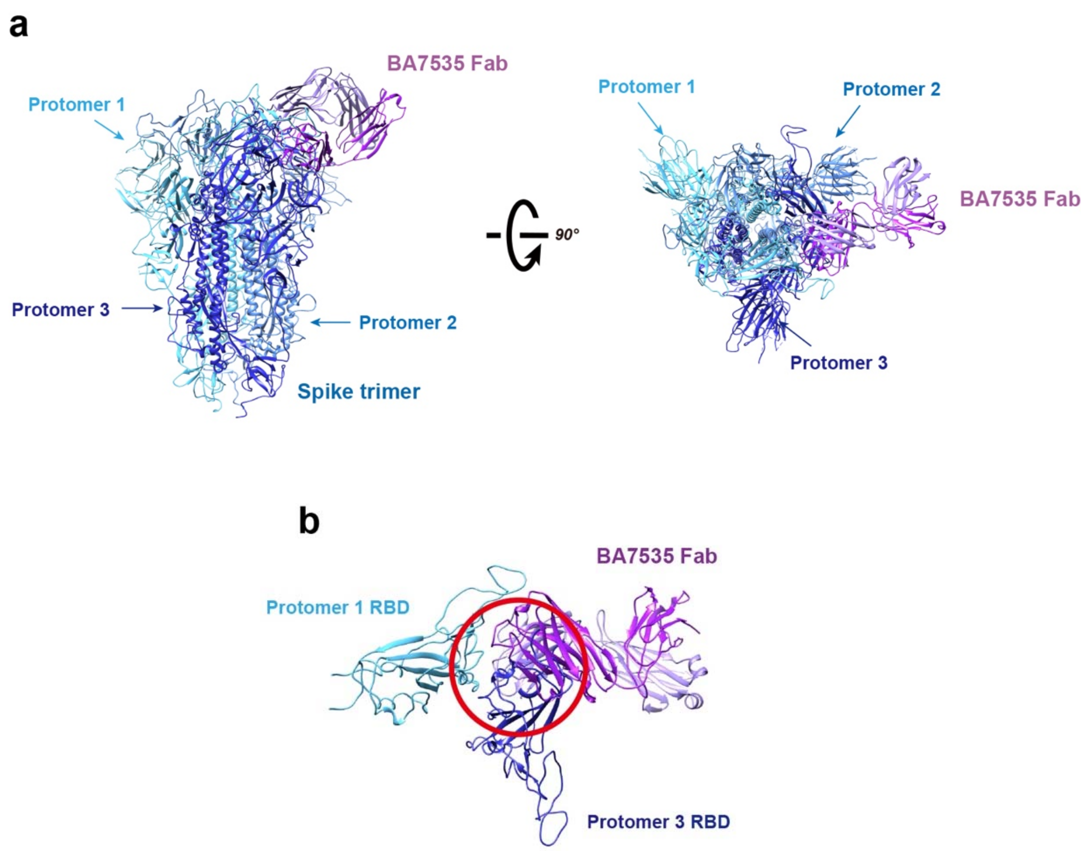

Supplementary Figure. 8. The binding site of BA7535 is partially blocked by the adjacent RBD when the RBDs are in the 'down' conformation.

(a) Structure of spike trimer with 3 RBDs in the 'down' conformation, with one BA7535-Fab displayed binding onto the protomer 1. **(b)** Zoomed-in view of BA7535-Fab binding onto the protomer 1 RBD, however, the binding site is partially blocked by the RBD from adjacent protomer 3 RBD (highlighted in red circle).

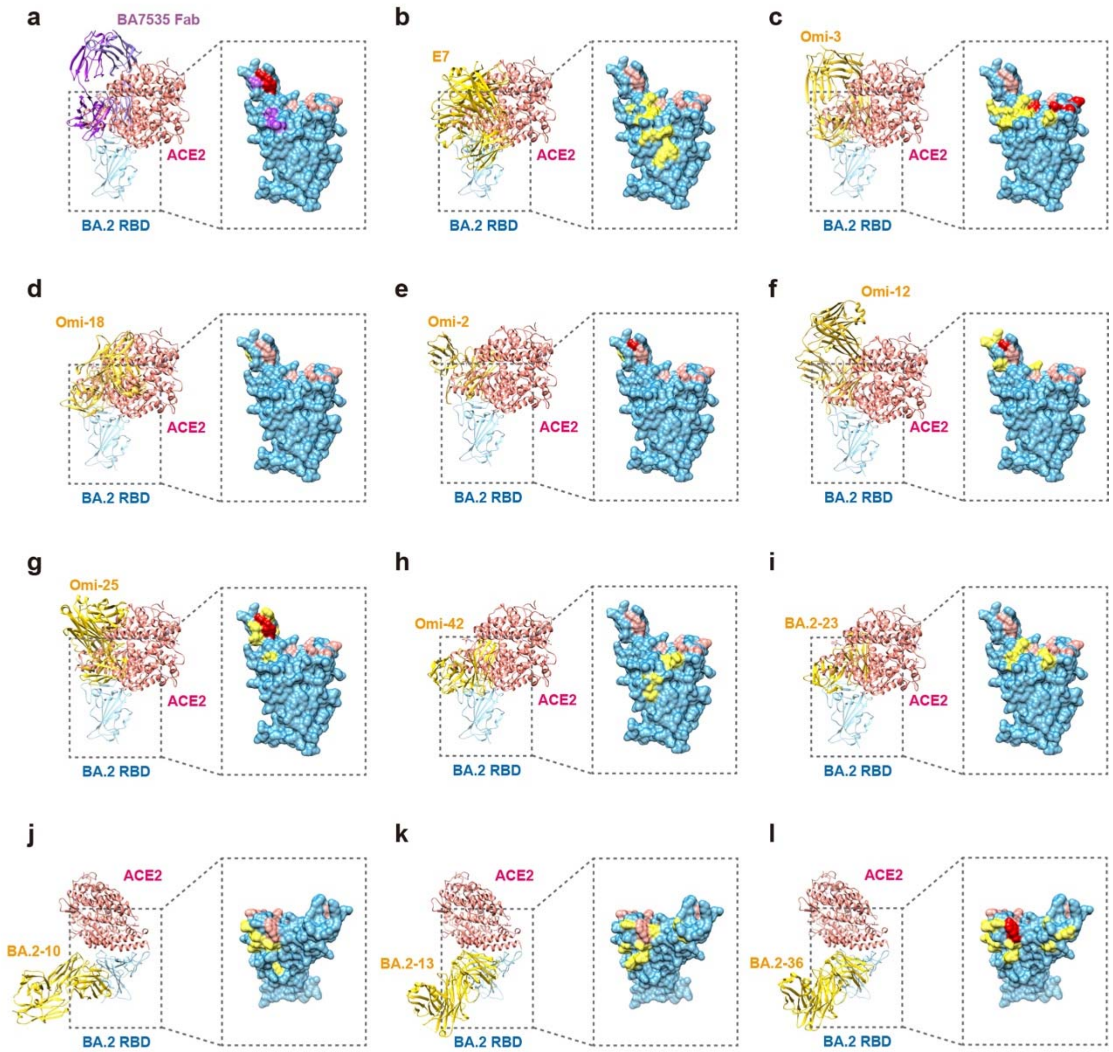

Supplementary Figure. 9. BA7535 binding mode analysis compared to other antibodies.

The complex of BA.2 RBD (cyan) and ACE2 (pink) is superimposed with antibodies displayed in as ribbon, and in the zoomed-views, the binding site of ACE2 (pink) and epitopes of each antibody (purple for BA7535 and yellow for other antibodies) are highlighted on the surface of RBD structure (cyan), with the epitopes that overlap with ACE2 binding site shown in red. **(a)** BA7535-Fab (purple), **(b)** E7 (yellow), **(c)** Omi-2(yellow), **(d)** Omi-12 (yellow), **(e)** Omi-3 (yellow), **(f)** Omi-18 (yellow), **(g)** Omi-25 (yellow), **(h)** Omi-42 (yellow), **(i)** BA.2-23 (yellow), **(j)** BA.2-10 (yellow), **(k)** BA.2-13 (yellow), **(l)** BA.2-36 (yellow).

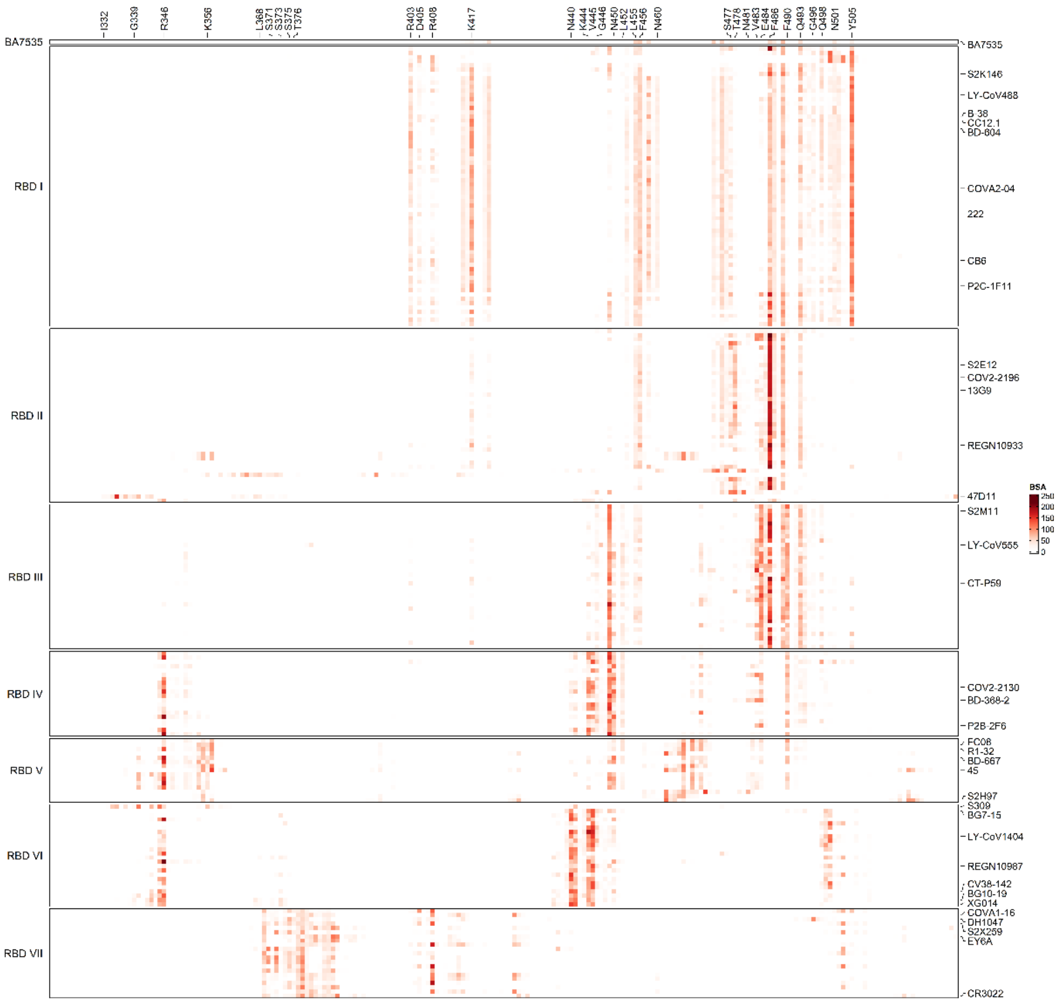

Supplementary Figure. 10. Comparative analysis of epitopes of BA7535 and 221 published RBD-specific mAbs. A total of 221 human SARS-CoV-2 RBD-specific mAbs with available structures were downloaded from the PDB (https://www.rcsb.org/). The PDB accession codes for the 221 mAbs were summarized in Supplementary Table 4. Epitope residues and buried surface area (BSA) for each epitope residue of the 221 RBD-specific mAbs as well as BA7535 were determined using the PDBePISA server (https://www.ebi.ac.uk/msd-srv/prot_int/). BSA for each epitope residue is considered a feature of a certain antibody and used to construct a feature matrix MAxB for downstream analysis, where A is the number of antibodies and B is the number of features (amino acid length of RBD: V320-K529). After the dimensionality reduction analysis, the 221 mAbs were clustered into seven groups, namely RBD I-VII. The BSA for each epitope residue of the 221 RBD-specific mAbs as well as BA7535 were shown

as heatmap using the R package ComplexHeatmap (v 2.14.0). Source data are provided as a Source Data file.

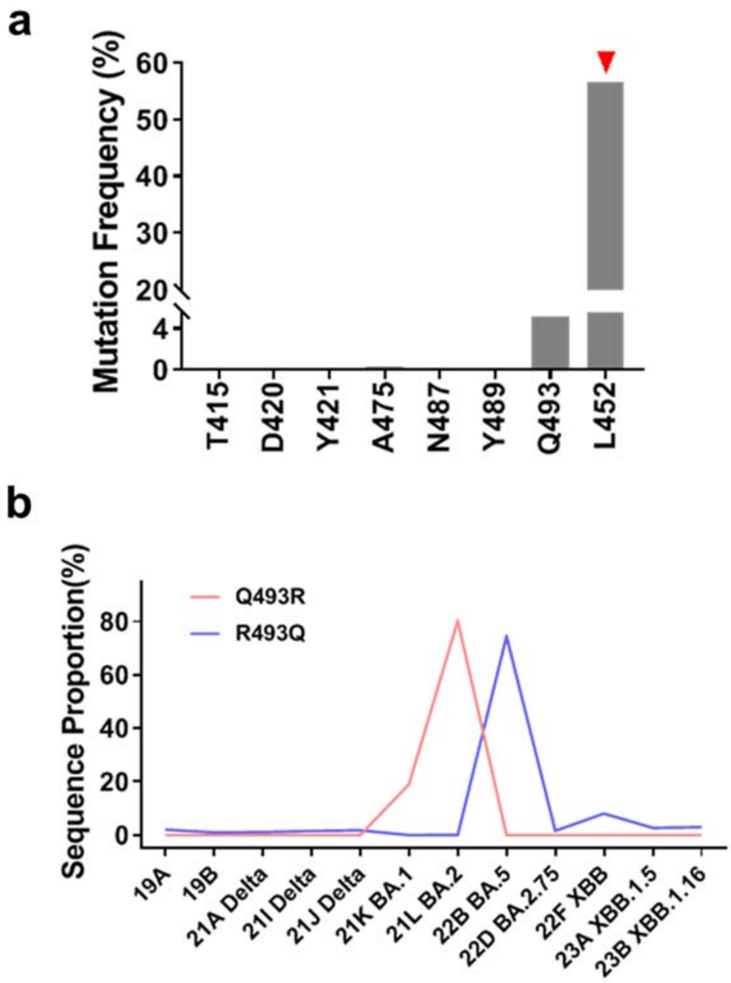

Supplementary Figure. 11. Mutation frequency of BA7535-interacting residues based on GISAID dataset. L452 was indicated in pink on RBD of Omicron and set as a control. **(a)** mutation frequency of BA7535-interacting residues, **(b)** mutation frequency of Q493R and R493Q. Source data are provided as a Source Data file.

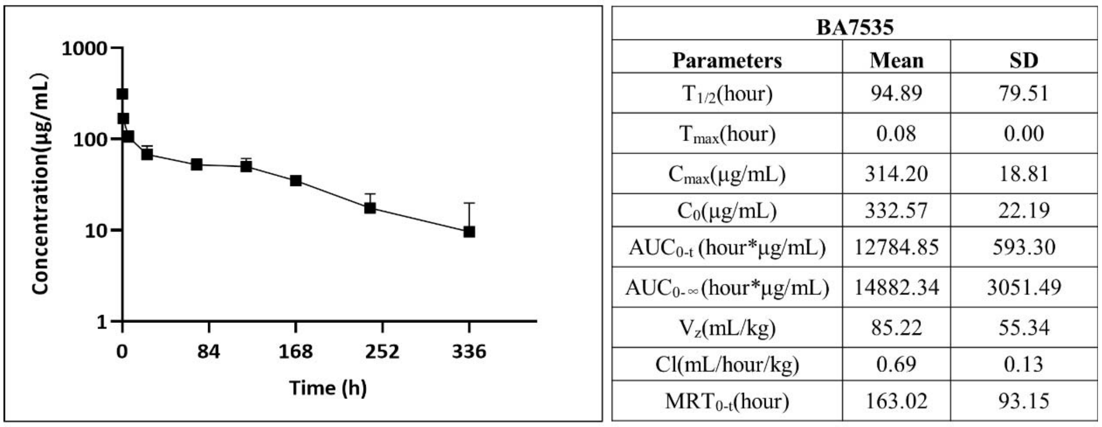

BA7535		
Parameters	Mean	SD
$T_{1/2}$(hour)	94.89	79.51
T_{max}(hour)	0.08	0.00
C_{max}(μg/mL)	314.20	18.81
C_0(μg/mL)	332.57	22.19
AUC_{0-t} (hour*μg/mL)	12784.85	593.30
$AUC_{0-\infty}$(hour*μg/mL)	14882.34	3051.49
V_z(mL/kg)	85.22	55.34
Cl(mL/hour/kg)	0.69	0.13
MRT_{0-t}(hour)	163.02	93.15

Supplementary Figure. 12. Pharmacokinetic of BA7535 in BALB/c mice. Three healthy BALb/c mice were administered intravenously with BA7535 at a single dose of 10 mg/kg. Enzyme-linked immunosorbent assay (ELISA) was used to determine the concentration of BA7535 in serum. ELISA experiment was performed once. The main PK kinetic parameters were calculated using Phoenix WinNonlin software. Data were presented as mean and SD. Source data are provided as a Source Data file.

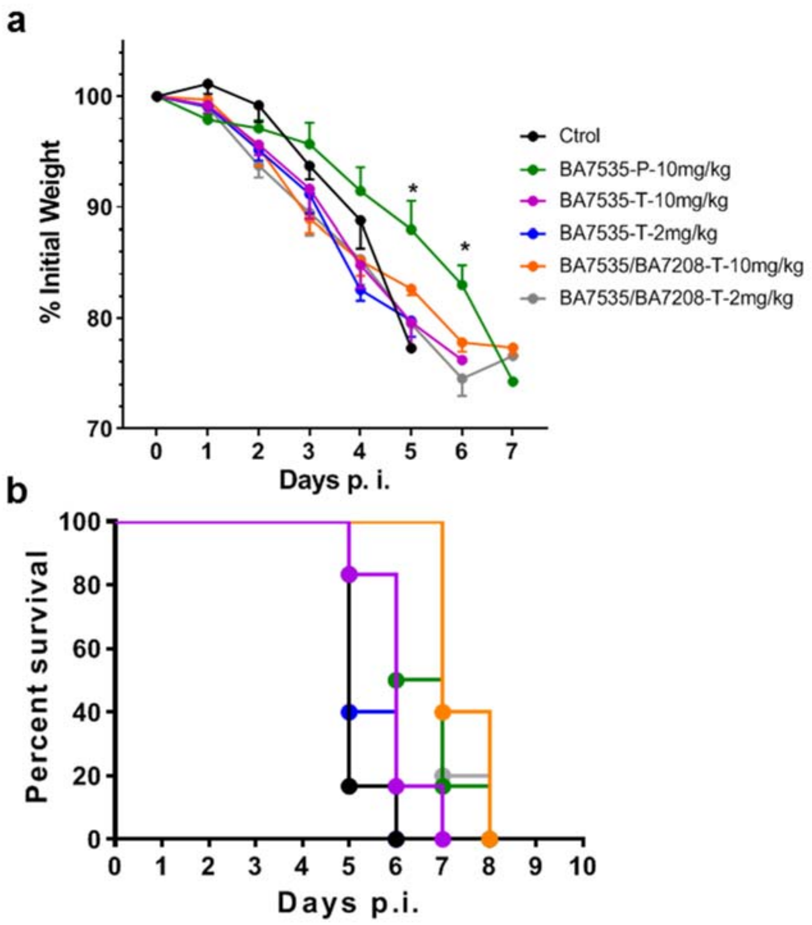

Supplementary Figure. 13. Weight loss and survival rate was monitored. Prophylactic and therapeutic tests of BA7535 and BA7535/BA7208 against BA.5 in hACE2 transgenic mice (n=5), Antibody was administrated to hACE2 transgenic mice intraperitoneally (i.p) 24 h before (prophylactic, abbreviation P) or 8 h after (therapeutic, abbreviation T) SARS-CoV-2 Omicron BA.5 infection (1×10^5 FFU, intranasally). symbols represent the mean ± SEM. Source data are provided as a Source Data file.

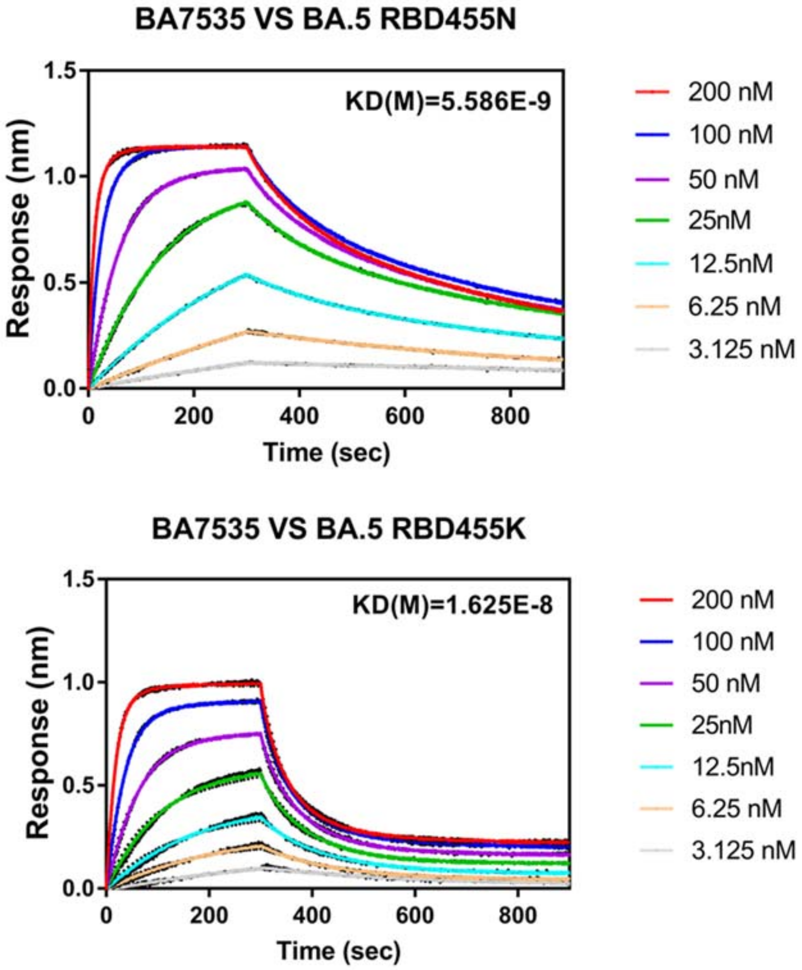

Supplementary Figure. 14. Comparison of binding affinity between BA7535 VS BA.5 RBD455N and BA7535 VS BA.5 RBD455K. Source data are provided as a Source Data file.

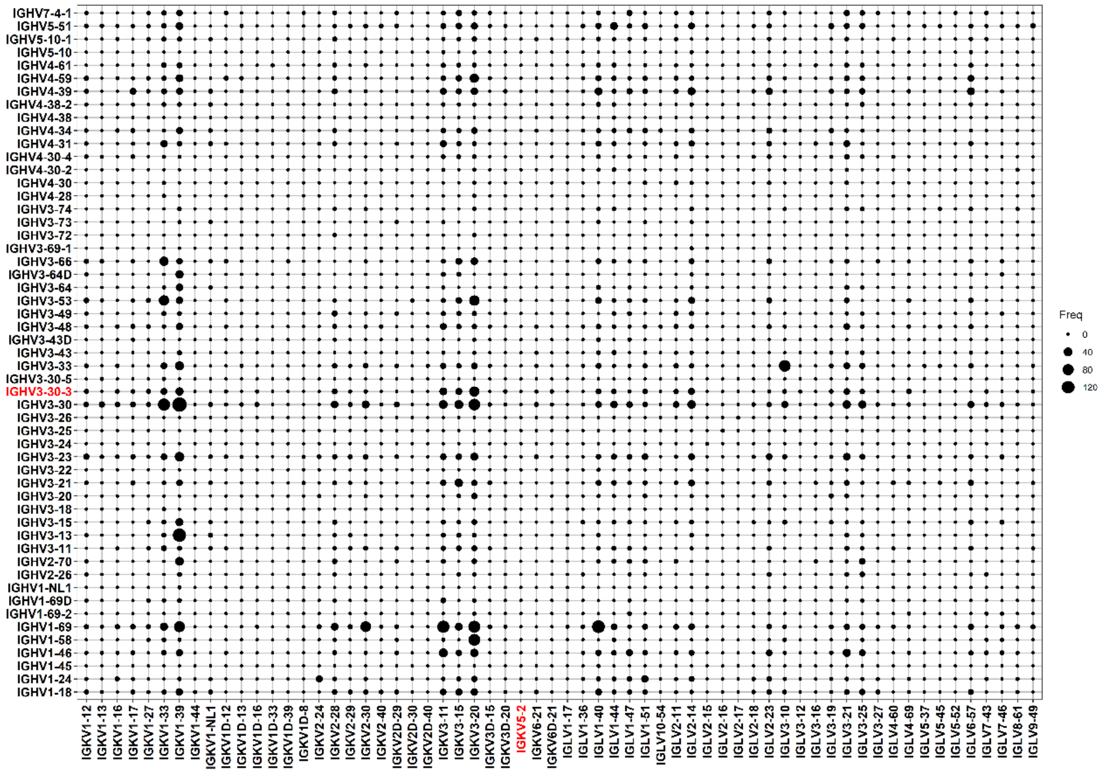

Supplementary Figure. 15. Germline usage analysis of 5712 SARS-CoV-2 spike-specific human mAbs from the COV-AbDab database. BA7535 is derived from the pairing of IGHV3-30-3 and IGKV5-2. Source data are provided as a Source Data file.

Supplementary Table 1. Cryo-EM data collection, refinement, and validation statistics for the structure of Omicron BA.2 Spike with BA7535 Fab

Omicron BA.2 RBD-BA7535 Consensus map	
**Data collection and processing**	
Magnification	130k
Voltage (kV)	300
Electron exposure (e^{-}/Å^{2})	50
Defocus range (μm)	-1.2 to -2.0
Pixel size (Å)	0.668
Symmetry imposed	C1
Final particle images (no.)	920863
Map resolution (Å)	2.40
FSC threshold	0.143
Map resolution range (Å)	2.0-6.5
**Refinement**	
Initial model used (PDB code)	7XDL
Model composition	
Non-hydrogen atoms	34380
Protein residues	4356
Ligands	36
R.m.s. deviations	
Bond lengths (Å)	0.003
Bond angles (°)	0.596
Validation	
MolProbity score	1.49
Clashscore	5.21
Poor rotamers (%)	0.00
Ramachandran plot	
Favored (%)	96.62
Allowed (%)	3.35
Disallowed (%)	0.02

Supplementary Table 2. Protein-protein interaction between Omicron BA.2 RBD and BA7535-Fab

	Residue in Omicron BA.2 RBD	Residue in BA7535 Fab	Distance (Å)
Hydrogen bond	Thr(T)-415	Tyr(Y)-106	3.1
	Asp(D)-420	Tyr(Y)-106	2.4
	Tyr(Y)-421	Leu(L)-103	3.6
	Ala(A)-475	Thr(T)-28 (main chain)	3.6
	Ala(A)-475	Thr(T)-28 (side chain)	3.4
	Asn(N)-487	Arg(R)-98	3.6
	Tyr(Y)-489	Arg(R)-98	3.5
Salt bridge	Arg(R)-493	Glu(E)-50	3.8

Residues in light purple cells are in light chain of BA7535 Fab, Residues in dark purple cells are in heavy chain of BA7535 Fab. A salt bridge is formed between the side chains of R493 and E50, with both the distances between the carboxylic OE1 atom of E50 and the guanidinium NH1/NH2 atoms of R493 of 3.8 Å, as illustrated in Fig. 3b.

Supplementary Table 3. Impact of Omicron BA.2/antibody binding interface.

Complex	Interface area, Å^2	ΔG kcal/mol	N_{HB}	N_{SB}
**BA7535-BA.2**	722	-6.3 (HC)<br>0.4 (LC)	6	1
**LY-Cov555-BA.2**	1217	-7.4 (HC)<br>-2.9 (LC)	6	0
**LY-Cov016-BA.2**	1026	-1.3 (HC)<br>-0.4 (LC)	11	0
**A23-58.1-BA.2**	611	-2.7 (HC)<br>-1.8 (LC)	4	0
**REGN10933-BA.2**	916	-0.5 (HC)<br>-2.1 (LC)	5	1
**2196-BA.2**	749	-4.4 (HC)<br>-1.6 (LC)	7	0
**SA55-BA.2**	842	-8.3 (HC)<br>-0.9 (LC)	7	0
**2130-BA.2**	762	-6.3 (HC)<br>-4.5 (LC)	4	0
**LY-Cov1404-BA.2**	789	-8.2 (HC)<br>-1.1 (LC)	5	0
**REGN10987-BA.2**	577	-3.8 (HC)<br>-2.5 (LC)	4	1
**Vir7831-BA.2**	886	-4.5 (HC)<br>-3.8 (LC)	7	2
**S309-BA.2**	693	-8.4 (HC)<br>-1.8 (LC)	3	0
**SA58-BA.2**	799	-3.4 (HC)<br>-3.6 (LC)	7	0
**BA7208-BA.2**	599	-4.4 (HC)<br>-1.2 (LC)	3	1

Supplementary Table 4. Summary and classification of 221 RBD-specific mAbs with available structures from the PDB

No.	mAb name	Epitope	PBD ID
1	C102	RBD I	7K8M
2	S2H14	RBD I	7JX3
3	CB6	RBD I	7C01
4	CV07-250	RBD I	6XKQ
5	C105	RBD I	6XCM/6XCN
6	B-38	RBD I	7BZ5
7	COVA2-04	RBD I	7JMO
8	CC12.1	RBD I	6XC2/6XC3
9	CC12.3	RBD I	6XC4/6XC7
10	BD-236	RBD I	7CHB
11	BD-604	RBD I	7CH4
12	BD-629	RBD I	7CH5
13	C1A-B3	RBD I	7KFW
14	C1A-F10	RBD I	7KFY
15	C1A-C2	RBD I	7KFX
16	C1A-B12	RBD I	7KFV
17	P2C-1F11	RBD I	7CDI
18	CV30	RBD I	6XE1
19	150	RBD I	7ND5/7BEI
20	40	RBD I	7ND6/7ND3
21	88	RBD I	7ND4/7BEL
22	269	RBD I	7NEH/7BEM
23	158	RBD I	7BEJ/7BEK
24	910-30	RBD I	7KS9
25	P4A1	RBD I	7CJF
26	P5A-3A1	RBD I	7D0C
27	P5A-1B8	RBD I	7CZR/7CZS/7D00
28	P5A-1B6	RBD I	7CZV/7CZU
29	P2B-1A1	RBD I	7CZP
30	P2B-1A10	RBD I	7CZQ
31	P5A-2G9	RBD I	7CZT
32	LY-CoV488	RBD I	7KMH
33	LY-CoV481	RBD I	7KMI
34	222	RBD I	7NX6
35	STE90-C11	RBD I	7B3O

36	BG4-25	RBD I	7M6D
37	ab1	RBD I	7MJJ/7MJK/7MJL
38	BD-515	RBD I	7E88
39	BD-508	RBD I	7E86
40	P22A-1D1	RBD I	7CHS
41	P5A-3C8	RBD I	7CHP
42	P5A-1D2	RBD I	7CHO
43	2B11	RBD I	7E5Y
44	C099	RBD I	7R8L
45	C098	RBD I	7N3I
46	RBD-10D12	RBD I	7E3C
47	BD-813	RBD I	7EY0
48	NT-193	RBD I	7E5O
49	S-B8	RBD I	7KN3
50	S-E6	RBD I	7KN4
51	CoV11	RBD I	7S4S
52	PDI 42	RBD I	7MZG
53	PDI 37	RBD I	7MZF
54	PDI 210	RBD I	7MZL
55	PDI 231	RBD I	7MZN
56	E4	RBD I	7VMU
57	ION-360	RBD I	7NP1
58	MW07	RBD I	7DK2
59	S2K146	RBD I	7TAS/7TAT
60	FI-3A	RBD I	7Q0A/7PQZ
61	ADI-55688	RBD I	7U2E
62	ADG20	RBD I	7U2D
63	C98C7	RBD I	7SWO
64	BD-503	RBD I	7EJY
65	CV2.2325	RBD I	7QF0
66	C110	RBD II	7K8V
67	S2H13	RBD II	7JV4/7JV2/7JV6
68	REGN10933	RBD II	6XDG
69	P17	RBD II	7CWM/7CWL/7CWN/7CWO
70	253H165L	RBD II	7NDB
71	253H55L	RBD II	7ND9/7NDA/7BEO
72	253	RBD II	7BEN
73	H4	RBD II	7L58
74	S2E12	RBD II	7K45/7K4N

75	15033-7	RBD II	7KLH
76	15033	RBD II	7KLG
77	P5A-3C12	RBD II	7D0B/7D0D
78	P5A-2F11	RBD II	7CZY/7CZZ
79	47D11	RBD II	7AKJ/7AKD
80	PR953	RBD II	7DEU
81	298	RBD II	7K9Z
82	BD-623	RBD II	7E7Y
83	A23-58.1	RBD II	7LRS/7LRT
84	B1-182.1	RBD II	7MLZ/7MM0
85	COV2-2196	RBD II	7L7E
86	S-7B8	RBD II	7E39
87	BD-836	RBD II	7EZV
88	BD-771	RBD II	7EY5
89	CV503	RBD II	7LQ7
90	58G6	RBD II	7E3L
91	13G9	RBD II	7E3K
92	C118	RBD II	7RKS/7RKV
93	PDI 222	RBD II	7RR0
94	WCSL 129	RBD II	7MZI/7MZK/7MZJ
95	WCSL 119	RBD II	7MZH
96	P5C3	RBD II	7P40/7NY5
97	clone 2	RBD II	7MW5/7MW6
98	2G1	RBD II	7X08
99	CV07-287	RBD II	7S5R
100	XGv347	RBD II	7WED/7WEA/7WEB/7WEC
101	G32A4	RBD II	7SWN
102	87G7	RBD II	7R40
103	J08	RBD II	7SBU
104	CV2.6264	RBD II	7QF1
105	CV2.1169	RBD II	7QF7
106	UT28K	RBD II	7X7O
107	C002	RBD III	7K8S/7K8T
108	C121	RBD III	7K8X/7K8Y
109	C144	RBD III	7K90
110	CT-P59	RBD III	7CM4
111	COVA2-39	RBD III	7JMP
112	BD-23	RBD III	7BYR
113	P2C-1A3	RBD III	7CDJ

114	316	RBD III	7ND7/7BEH
115	384	RBD III	7ND8/7BEP
116	2-15	RBD III	7L5B
117	2-43	RBD III	7L56
118	2-4	RBD III	6XEY
119	DH1041	RBD III	7LAA
120	DH1043	RBD III	7LJR
121	S2M11	RBD III	7K43/7K3Q
122	CV05-163	RBD III	7LOP
123	P5A-2G7	RBD III	7D03/7CZW
124	P5A-1B9	RBD III	7CZX
125	LY-CoV555	RBD III	7L3N
126	5A6	RBD III	7KQB/7M71
127	CA521	RBD III	7E23
128	BG7-20	RBD III	7M6H
129	BG1-24	RBD III	7M6I
130	PR1077	RBD III	7DEO
131	MW05	RBD III	7DK0
132	MW01	RBD III	7DJZ
133	REGN10989	RBD III	7M42
134	S2D106	RBD III	7R7N
135	C051	RBD III	7R8N
136	C548	RBD III	7R8O
137	nCoV617	RBD III	7E3O
138	WRAIR-2173	RBD III	7N4J
139	clone 6	RBD III	7MW4
140	DH1042	RBD III	7THT/7THE
141	C119	RBD IV	7K8W
142	CV07-270	RBD IV	6XKP
143	P2B-2F6	RBD IV	7BWJ
144	BD-368-2	RBD IV	7CHH
145	75	RBD IV	7BEN/7BEO
146	1-57	RBD IV	7LS9
147	47D1	RBD IV	7MF1
148	278	RBD IV	7OR9
149	RBD-9G11	RBD IV	7E3B
150	COV2-2130	RBD IV	7L7E
151	BD-804	RBD IV	7EYA
152	N-612-056	RBD IV	7S0B

153	PDI 93	RBD IV	7MZJ
154	JMB2002	RBD IV	7WPD/7WPF
155	FD-5D	RBD IV	7PR0
156	XGv282	RBD IV	7WLC/7WE7
157	A19-46.1	RBD IV	7TC9/7U0D/7TCC/7TCA/
158	A19-61.1	RBD IV	7TBF/7TB8
159	Fab06	RBD IV	7WPH
160	P36-5D2	RBD IV	7FAF/7FAE
161	45	RBD V	7BEL
162	FC08	RBD V	7DX4
163	S2H97	RBD V	7M7W
164	52	RBD V	7K9Z
165	R1-32	RBD V	7YDI
166	G32R7	RBD V	7N64
167	BD-744	RBD V	7EY0
168	BD-667	RBD V	7EY4
169	FD20	RBD V	7CYV/7URQ/7URS
170	N-612-017	RBD V	7S0C
171	PDI 215	RBD V	7MZM
172	WRAIR-2057	RBD V	7N4I
173	ION-300	RBD V	7BNV
174	58	RBD V	7QNY
175	35B5	RBD V	7E9N/7F46/7E9P
176	C135	RBD VI	7K8Z
177	S309	RBD VI	6WPS/6WPT
178	REGN10987	RBD VI	6XDG
179	2-7	RBD VI	7LSS
180	CV38-142	RBD VI	7LM8/7LM9
181	BG7-15	RBD VI	7M6G
182	BG10-19	RBD VI	7M6E
183	PR961	RBD VI	7DET
184	LY-CoV1404	RBD VI	7MMO
185	C032	RBD VI	7R8M
186	BD-812	RBD VI	7EZV
187	BD-821	RBD VI	7EY5
188	PDI 96	RBD VI	7MZK
189	XG005	RBD VI	7V26
190	XG014	RBD VI	7V2A
191	FD-11A	RBD VI	7PQZ

192	A5-10	RBD VI	7F7E
193	XGv289	RBD VI	7WEF/7WE9
194	XGv265	RBD VI	7WEE/7WE8
195	54042-4	RBD VI	7T01
196	32C7	RBD VI	7EGN/7ENF
197	ZWC6	RBD VI	7WWM
198	ZWD12	RBD VI	7WWL
199	510A5	RBD VI	7WS6
200	S2A4	RBD VII	7JVA/7JVC
201	S304	RBD VII	7JW0
202	EY6A	RBD VII	6ZDH
203	CR3022	RBD VII	6W41
204	DH1047	RBD VII	7LD1/7SG4
205	COVA1-16	RBD VII	7JMW
206	3D11	RBD VII	7M7B/7KQE
207	S2X259	RBD VII	7M7W/7RAL/7RA8
208	CV2-75	RBD VII	7M3I
209	MW06	RBD VII	7DPM
210	REGN10985	RBD VII	7M42
211	S2X35	RBD VII	7R6W
212	C022	RBD VII	7RKU
213	GH12	RBD VII	7D6I
214	2-36	RBD VII	7N5H
215	C1C-A3	RBD VII	7SN2/7SN3
216	AB-3467	RBD VII	7MSQ
217	G32Q4	RBD VII	7SWP
218	10-28	RBD VII	7SI2
219	10-40	RBD VII	7SD5
220	GW01	RBD VII	7EPX
221	STS165	RBD VII	7XIC/7XID

Supplementary Table 5. Mutations from 42 Omicron sub-lineages

Pango lineages	Mutations in spike compared to BA.2
BA.2	--
BA.2.3.20	K444R+N450D+L452M+N460K+R493Q
BA.2.10.4	G446S+F486P+R493Q+S494P
BJ.1	D339H+R346T+L368I+V445P+G446S+V483A+F490V
XBB.1	D339H+R346T+L368I+V445P+G446S+N460K+F486S+F490S+R493Q
XBB.1.5	D339H+R346T+L368I+V445P+G446S+N460K+F486L+F490S+R493Q
BA.2.38.1	N417T+K444N
BA.2.74	R346T+L452M
BA.2.76	R346T
BA.2.77	K356R+L452R+E340K
BA.2.79	N450D
BA.2.80	R346T+K356R+E340K
BA.2.75	G339H+G446S+N460K+R493Q
BL.1	G339H+R346T+G446S+N460K+R493Q
BR.1	G339H+G446S+N460K+R493Q+L452R+K444M
BN.2.1	G339H+G446S+N460K+R493Q+K356T+F490S
BN.1	G339H+G446S+N460K+R493Q+R346T+K356T+F490S
BA.2.75.2	G339H+G446S+N460K+R493Q+R346T+F486S
BM.1.1	G339H+G446S+N460K+R493Q+R346T+F486S
BM.1.1.1	G339H+G446S+N460K+R493Q+R346T+F486S+F490S
BR.2	G339H+G446S+N460K+R493Q+R346T+L452R+F486I
CA.1	G339H+G446S+N460K+R493Q+R346T+L452R+F486S
BA.2.75.4	G339H+G446S+N460K+R493Q+L452R
BA.2.75.5	G339H+G446S+N460K+R493Q+K356T
BM.1	G339H+G446S+N460K+R493Q+F486S

BA.4/5	L452R+F486V+R493Q
BA.4.6.1	L452R+F486V+R493Q+R346T
BA.5.6.2	L452R+F486V+R493Q+K444T
BQ.1	L452R+F486V+R493Q+K444T+N460K
BU.1	L452R+F486V+R493Q+K444M+N460K
BQ.1.1	L452R+F486V+R493Q+R346T+K444T+N460K
BF.16	L452R+F486V+R493Q+K444R
BA.4.6	L452R+F486V+R493Q+R346T
BA.4.7	L452R+F486V+R493Q+R346S
BA.5.9	L452R+F486V+R493Q+R346I
BA.5.5.1	L452R+F486V+R493Q+N450D
BA.5.2.7	L452R+F486V+R493Q+K444M
BA.5.1.12	L452R+F486V+R493Q+V445A
BF.7	L452R+F486V+R493Q+R346T
XBB.1.16	D339H+R346T+L368I+V445P+G446S+N460K+K478R +F486P+F490S+R493Q
XBB.1.9.1	D339H+R346T+L368I+V445P+G446S+N460K+F486S+ F490S+R493Q
EG.5	D339H+R346T+L368I+V445P+G446S+F456L+N460K+ F486P+F490S+R493Q

Supplementary Table 6. Impact of Omicron sub-lineages mutation on RBD/BA7535-Fab binding interface.

	Interface area (Å^2)	N_{HB}	N_{SB}
**WT (BA.2)**	722	6	1
**F486V**	710	6	1
**F486S**	696	6	1
**F486I**	716	6	1
**F486P**	696	6	1
**N417T**	761	6	1
**R493Q**	742	5	0
