# Supplementary material for: Identification of a highly conserved neutralizing epitope within the RBD region of diverse SARS-CoV-2 variants
Source: Nat Commun. 2024 Jan 29;15:842. doi: 10.1038/s41467-024-45050-3 (PMC10825162; doi:10.1038/s41467-024-45050-3)
Supplement: Supplementary file 3 — Reporting Summary [file 41467_2024_45050_MOESM3_ESM.pdf]

nature portfolio

Corresponding author(s): Jincun Zhao, Zheng Liu, Changlin Dou, Lan Wang

Last updated by author(s): Dec 28, 2023

Reporting Summary

Nature Portfolio wishes to improve the reproducibility of the work that we publish. This form provides structure for consistency and transparency in reporting. For further information on Nature Portfolio policies, see our Editorial Policies and the Editorial Policy Checklist.

Statistics

For all statistical analyses, confirm that the following items are present in the figure legend, table legend, main text, or Methods section.

n/a	Confirmed	
☐	☒	The exact sample size (*n*) for each experimental group/condition, given as a discrete number and unit of measurement
☐	☒	A statement on whether measurements were taken from distinct samples or whether the same sample was measured repeatedly
☐	☒	The statistical test(s) used AND whether they are one- or two-sided<br>*Only common tests should be described solely by name; describe more complex techniques in the Methods section.*
☒	☐	A description of all covariates tested
☒	☐	A description of any assumptions or corrections, such as tests of normality and adjustment for multiple comparisons
☐	☒	A full description of the statistical parameters including central tendency (e.g. means) or other basic estimates (e.g. regression coefficient) AND variation (e.g. standard deviation) or associated estimates of uncertainty (e.g. confidence intervals)
☐	☒	For null hypothesis testing, the test statistic (e.g. *F*, *t*, *r*) with confidence intervals, effect sizes, degrees of freedom and *P* value noted<br>*Give P values as exact values whenever suitable.*
☒	☐	For Bayesian analysis, information on the choice of priors and Markov chain Monte Carlo settings
☒	☐	For hierarchical and complex designs, identification of the appropriate level for tests and full reporting of outcomes
☒	☐	Estimates of effect sizes (e.g. Cohen's *d*, Pearson's *r*), indicating how they were calculated

Our web collection on statistics for biologists contains articles on many of the points above.

Software and code

Policy information about availability of computer code

Data collection	Absorbance (ELISA) and luminescence (pseudotype neutralizing assay) were meaured by Bioteck Synergy HT Multi-detection reader.<br>The number of SARS-CoV-2 foci was calculated using an EliSpot reader (Cellular Technology Ltd.).<br>SPR was performed by BIAcore 8K system.<br>ADCC and ADCP reporter bioassay were conducted by Tecan Microplate Reader.<br>Beam-induced motion correction was performed on stack of frames using MotionCor2. Initial contrast transfer function (CTF) values for each micrograph were calculated with CTFFIND4.<br>Cryo-EM micrographs were collected on a 300kV Thermo Fisher Scientific Titan Krios G3i electron microscope equipped with a GIF-Quantum energy filter (Gatan).<br>Images were recorded with Gatan K3 direct electron detector.<br>We used Excel 97-2003 for the data collection.<br>Flow cytometry was done by NovoCyte 2060R flow cytometry.
Data analysis	Bio-Layer Interferometry (BLI) data was processed using ForteBio's Data Analysis Software 9.0.<br>Antibody binding kinetics and competition analysis were analyzed by with BIAevaluation software<br>Statistical analysis was performed using Graphpad prism 8.0<br>Antibody sequence alignment was performed by BioEdit V7.2.<br>H&E imagine were process by iViewer software.<br>The main PK kinetic parameters were calculated using Phoenix WinNonlin.<br>The model of Omicron BA.2 S-BA7535 complex was built based on the model of Delta Spike protein in complex with BA7208-Fab and BA7125-Fab (PDB:7XDL). The model was manually modified in COOT.<br>The modified model was refined using phenix.realspace_refine program in PHENIX software package.

Flow Cytometry data were analyzed by FlowJo V10.
Local resolution estimate was performed with cryoSPARC.
CLC Genomics Workbench Version 21 was used for SNPs analysis.
Micrographs were selected for further data processing and reconstruction using cryoSPARC.

For manuscripts utilizing custom algorithms or software that are central to the research but not yet described in published literature, software must be made available to editors and reviewers. We strongly encourage code deposition in a community repository (e.g. GitHub). See the Nature Portfolio guidelines for submitting code & software for further information.

Data

Policy information about availability of data

All manuscripts must include a data availability statement. This statement should provide the following information, where applicable:

- Accession codes, unique identifiers, or web links for publicly available datasets
- A description of any restrictions on data availability
- For clinical datasets or third party data, please ensure that the statement adheres to our policy

All relevant data are available in the article, supplementary Information, or from the corresponding author J.Z. upon reasonable request. Cryo-EM density maps of the spike-Fab complex have been deposited in the Electron Microscopy Data Bank (EMD-34522 and EMD-34526). The atomic coordinates of above complexes have been deposited in the Protein Data Bank under the accession code 8H7L and 8H7Z, respectively. The sequences of BA7535 have been deposited in GenBank with the accession codes OP831943 and OP831944 for heavy chain and light chain respectively. The other source data generated in this study are provided in the Supplementary Information and the Source Data file. Source data are provided with this paper.

Research involving human participants, their data, or biological material

Policy information about studies with human participants or human data. See also policy information about sex, gender (identity/presentation), and sexual orientation and race, ethnicity and racism.

Reporting on sex and gender	No human participants were involved in this study
Reporting on race, ethnicity, or other socially relevant groupings	No human participants were involved in this study
Population characteristics	No human participants were involved in this study
Recruitment	No human participants were involved in this study
Ethics oversight	No human participants were involved in this study

Note that full information on the approval of the study protocol must also be provided in the manuscript.

Field-specific reporting

Please select the one below that is the best fit for your research. If you are not sure, read the appropriate sections before making your selection.

☒ Life sciences ☐ Behavioural & social sciences ☐ Ecological, evolutionary & environmental sciences

For a reference copy of the document with all sections, see nature.com/documents/nr-reporting-summary-flat.pdf

Life sciences study design

All studies must disclose on these points even when the disclosure is negative.

Sample size

Six human antibody transgenic mice BA-huMab were immunized with recombinant RBD protein of SARS-CoV-2 BA.1 for three rounds and one final boost in 10-day intervals. Freund complete adjuvant was used in 1st round, Freund incomplete adjuvant was used in 2nd-3th rounds and no adjuvant was used in the final booster. Spleen cells were harvested after three days of the last boost for phage libraries construction (Supplementary Figure. 1).

six- to eight-weeks old hACE2 transgenic mice were intraperitoneally injected with 2 or 10 mg/kg of BA7535 or BA7535/BA7208 cocktail per mouse 24 h before or 8 h after the challenge with 1×105 FFU SARS-CoV-2 Omicron BA.5 (n=3 mice/group). Mice injected with phosphate-buffered saline (PBS) were challenged with the same dose of SARS-CoV-2 as control. To investigate the presence of SARS-CoV-2 in the lungs and brains, lungs and brains were harvested for viral titers 2 and 4 day later by using focus forming assay (FFA) (Figure 5a-c).

The prophylactic and therapeutic efficacies of BA7535 against SARS-CoV-2 Omicron XBB.1 were evaluated in BALB/c mice, six- to eight-weeks old BALB/c mice (n=4 mice/group) were administered via intraperitoneally (i.p), intranasal (i.n), aerosol inhalation (a.i) with BA7535 per mouse 24 h before or after the challenge with 1×105 FFU SARS-CoV-2 Omicron XBB.1. Mice injected with PBS were challenged with the same dose of SARS-CoV-2 as control. Lungs were harvested for viral titers 2 days later by using focus forming assay (FFA) (figure 5d-g)

	A single intravenous injection of BA7535 was conducted in BALB/c mice (N=3, 3/group, female, age 7-8 weeks, body weight 20±1 g) at 10 mg/kg. Blood samples were collected at predose and 5 min, 1 h, 6 h, 24 h, 72 h, 120 h, 168 h, 240 h, 336 h postdose from the mice via orbit vein bleeding(Supplementary Figure. 12)
Data exclusions	No data were excluded
Replication	All experiments were repeated in at least duplicates except indicated. Similar findings were obtained from all repeats.
Randomization	There was no allocation except for the grouping of the animals. Animals were randomly allocated to the groups.
Blinding	No blinding was done. Blinding was not relevant to the study because the results are quantitative and objective, and does not require a subjective judgment.

Reporting for specific materials, systems and methods

We require information from authors about some types of materials, experimental systems and methods used in many studies. Here, indicate whether each material, system or method listed is relevant to your study. If you are not sure if a list item applies to your research, read the appropriate section before selecting a response.

Materials & experimental systems

n/a	Involved in the study
☐	☒ Antibodies
☐	☒ Eukaryotic cell lines
☒	☐ Palaeontology and archaeology
☐	☒ Animals and other organisms
☒	☐ Clinical data
☒	☐ Dual use research of concern
☒	☐ Plants

Methods

n/a	Involved in the study
☒	☐ ChIP-seq
☒	☐ Flow cytometry
☒	☐ MRI-based neuroimaging

Antibodies

Antibodies used	Goat Anti-Human IgG-HRP, SouthernBiotech,2049-05<br>HRP-conjugated Streptomycin,Beyotime, A0303<br>SARS-CoV/SARS-CoV-2 Nucleocapsid Rabbit PAb, Sino Biological, 40143-R001<br>Peroxidase AffiniPure Goat Anti-Rabbit IgG (H+L), Jackson, 111-035-144<br>Goat anti-Human IgG Fc, FITC, Thwemo Fisher Scientific,A18818
Validation	All secondary antibodies used are commercial antibodies reported by the manufacturer to be validated for use

Eukaryotic cell lines

Policy information about cell lines and Sex and Gender in Research

Cell line source(s)	African green monkey kidney Vero cell (ATCC, vero E6) were obtained from ATCC,<br>Huh-7 cells (0403) were obtained from JCRB;<br>CHOK1-Spike cell line (RD00819) was obtained from Genscript;<br>Jurkat-FcγRIIA-H131 Effector cells (DD1304-0) were obtained from Vazyme;<br>ADCC Bioassay Effector cells (G7011) were obtained from Promega.<br>Expi-CHO Expression system (Gibco,A29133)
Authentication	All cell lines were frequently checked for cellular morphologies, growth rates and functions. All purchased cell lines were available in commercial company.
Mycoplasma contamination	All cell lines used were tested (by PCR) and were mycoplasma free.
Commonly misidentified lines<br>(See ICLAC register)	No commonly misidentified lines were used in this study.

Animals and other research organisms

Policy information about studies involving animals; ARRIVE guidelines recommended for reporting animal research, and Sex and Gender in Research

Laboratory animals	six human antibody transgenic mice BA-huMab (6-8 week, female) were used for immunization.<br>Three healthy BALb/c mice were used for Pharmacokinetic analysis (BALB/c, 6-8 week female).<br>BALB/c (6-8 week female) mice were used for the prophylactic and therapeutic efficacy evalution of BA7535 against SARS-CoV-2

	Omicron XBB.1<br>hACE2 transgenic mice mice (K18-hACE mice, 6-8 weeks, female) were used for protection experiments.<br>All the animals were housed in an environment with a temperature of 22 ± 1 ºC, a relative humidity of 50 ± 1%, and a light/dark cycle of 12/12 hr.
Wild animals	No wild animals were used in the study.
Reporting on sex	female, 6-8 weeks
Field-collected samples	No field collected samples were used in the study.
Ethics oversight	All animal experiments were complied with relevant ethical regulations regarding animal research. Immunization and pharmacokinetics study procedures in mice were approved by the Institutional Animal Care Committee of Boan Biotech and the Approval Numbers are 2021-TS0001-36 and 2022-TS0001-21 respectively. The animal study was reviewed and approved by Institutional Animal Care and Use Committees of the First Affiliated Hospital of Guangzhou Medical University (2021-239).

Note that full information on the approval of the study protocol must also be provided in the manuscript.

Plants

Seed stocks	no
Novel plant genotypes	no
Authentication	no
